# Supplementary material for: Whole Exome Sequencing in Coloboma/Microphthalmia: Identification of Novel and Recurrent Variants in Seven Genes
Source: Genes (Basel). 2021 Jan 6;12(1):65. doi: 10.3390/genes12010065 (PMC7825129; doi:10.3390/genes12010065)
Supplement: Supplementary file 1 [file genes-12-00065-s001.pdf]

Table S1. Gene list used for filtering of WES data.

Gene associated with	Gene Symbol
a) **Syndromic MAC**†	*ACTB* [1], *ACTG1* [1,2], *AHI1* [3], *ALG3* [4], *ALX1* [5], *ALX3* [6], *ANOS1* [7], *ARX* [8], *B3GALNT2* [9], *B3GLCT* [10], *BCOR* [10], *BEST1* [10], *BMP4* [11], *BRPF1* [12], *C12orf57* [10], *CASK* [13], *CC2D2A* [10], *CCDC22* [14], *CDK9* [15], *CDON* [16,17], *CEP290* [4], *CHD7* [18], *CPLANE1* [10], *COL4A1* [19,20], *COX7B* [21], *CREBBP* [4], *CRIM1* [22], *CTU2* [23], *DAG1* [24], *DHX38* [25], *DPYD* [4], *DSC3* [26], *EFTUD2* [27], *EP300* [28], *ERCC1* [29], *ERCC5* [30], *ERCC6* [31], *EYA1* [10], *FADD* [4], *FAM111A* [32], *FANCA* [10], *FANCD2* [10], *FANCE* [33], *FANCI* [33], *FANCL* [33], *FAT1* [34], *FBXW11* [35], *FIBP* [36], *FKRP* [37], *FKTN* [10], *FLNA* [4], *FNBP4* [38], *FOXA2* [39], *FOXL2* [10], *FRAS1* [10], *FREM1* [10], *FREM2* [33,40], *GDF3* [10], *GDF6* [10], *GJA1* [41], *GRIP1* [10], *GZF1* [42], *HCCS* [10], *HESX1* [43], *HDAC6* [33], *HMGB3* [10], *HMX1* [10], *HRAS* [4], *IGBP1* [10], *IKBKG* [44], *INPP5E* [45], *INTS1* [46], *KCTD1* [4], *KDM6A* [13], *KERA* [10], *KIAA0586* [45], *KIAA1109* [47], *KIAA1279* [4,48], *KIF11* [49], *KIF26B* [50], *KMT2D* [51], *KRAS* [4], *LAMA1* [52], *LRP2* [4], *MAB21L2* [10], *MAPRE2* [53], *MED12L* [54], *MID1* [55], *MITF* [4], *MKS1* [4], *NAA10* [10], *NAGA* [56], *NDUFB11* [57], *NF1* [58], *NHS* [10], *NRAS* [4], *NTNG1* [59], *OFD1* [45], *OSGEP* [60], *OTX2* [10], *PAX2* [4], *PDE6D* [61], *PIGL* [62], *PITX2* [4], *PITX3* [10], *POC1B* [45], *POMGNT1* [63], *POMGNT2* [33], *POMK* [64], *POMT1* [65], *POMT2* [33], *PORCN* [4], *PQBP1* [4], *PTCH1* [4], *PTPN11* [4], *PUF60* [66], *RAB18* [67], *RAB3GAP1* [4], *RAB3GAP2* [10], *RARA* [68], *RARB* [10], *RAX* [10], *RERE* [69], *RIPK4* [70], *RPGRIP1L* [71], *SALL1* [4], *SALL4* [4], *SEMA3E* [72], *SH3PXD2B* [73], *SHH* [10], *SIX3* [4], *SLC18A2* [26], *SLC25A24* [74], *SLC38A8* [75], *SMARCA4* [76], *SMCHD1* [33], *SMG9* [77], *SMO* [78], *SMOC1* [10], *SOX2* [10], *SOX3* [79], *SOX10* [80], *SPINT2* [81], *SRD5A3* [82], *STRA6* [10], *TBC1D20* [83], *TBC1D32* [26,33], *TBX22* [84], *TCOF1* [10], *TFAP2A* [85], *TMEM67* [86], *TMEM216* [87], *TMEM237* [45], *TUBA1A* [88], *TUBB* [53], *TUBGCP4* [89], *TWF1* [2], *VAX1* [90], *VSX2* [10], *WASHC5* [91], *WDR11* [92], *WDR37* [93], *YAP1* [94], *ZEB2* [95], *ZMIZ1* [96]
b) **Non-syndromic MAC**‡	*ABCB6* [97], *ALDH1A3* [10], *ATOH7* [10], *CRYAA* [98], *CRYBA4* [10], *CRYBB1* [33], *CRYBB2* [33], *CRYGC* [33], *FOXC1* [4], *FOXE3* [10], *GJA8* [99,100], *IPO13* [101], *LCP1* [2], *MAF* [102], *MFRP* [33], *MYO10* [26], *NDP* [19], *OLFM2* [103], *PAX6* [10], *PIEZO2* [104], *PRSS56* [26], *PXDN* [10], *RBP4* [105,106], *SALL2* [10], *SIX6* [10], *TENM3* [107], *TMX3* [108]
c) **Animal models with MAC**	*ADAMTS16* [109], *ALDH7A1* [110], *BCL6* [111], *CAP2* [112], *CDH2* [113], *CDO1* [114], *FBN2* [115], *FGFR1* [116], *FGFR2* [116], *FOXG1* [117], *FRS2* [118], *GCLC* [119], *HES1* [120], *ISPD* [121], *JAG1* [122], *LAMB1* [123], *LAMC1* [123], *LMO2* [124], *LMX1B* [4], *MAPK8* [125], *MAPK9* [125], *NOG* [125], *PHACTR4* [126], *PPP1R12A* [127], *SFRP1* [128], *SFRP5* [128], *SMAD7* [129], *SOX4* [130], *SOX11* [131,132], *TGFB2* [133], *TLE3* [134], *VAX2* [4]
**Candidate Genes**	
d) **Genes coding for proteins of the SHH signalling pathway** [135]	*CER1*, *GLI2*, *GLI3*, *GREM1*, *SUFU*
e) **Genes coding for proteins of the WNT signalling pathway** [135]	*APC*, *CTNNB1*, *DVL1*, *FZD1*, *FZD2*, *FZD5*, *FZD6*, *FZD7*, *FZD9*, *FZD10*, *GSK3A*, *LRP5*, *LRP6*, *WNT1*, *WNT2*, *WNT2B*, *WNT3*, *WNT3A*, *WNT4*, *WNT5A*, *WNT5B*, *WNT6*, *WNT7A*, *WNT7B*, *WNT8A*, *WNT8B*, *WNT9A*, *WNT9B*, *WNT10A*, *WNT11*, *WNT16*

**f)**	**Additional candidate genes**	*AXIN2* [4], *BMP7* [136], *BMPR1A* [137], *BOC* [138], *CDK7* [139], *CENPH* [139], *CHD2* [135], *CRX* [135,140], *CRYAB* [135], *CRYBA1* [135], *CRYBA2* [135], *CRYBB3* [135], *CRYGA* [135], *CRYGB* [135], *CRYGD* [135], *CRYGS* [135], *CRYZ* [135], *CYP1B1* [141], *DIS3L2* [142], *DISP1* [143], *DKK1* [4], *DLX1* [135], *DLX2* [135], *EFNA5* [144], *EPHB2* [144], *FGF8* [145], *GAS1* [146], *GLI1* [147], *LHX1* [135], *NOD2* [148], *PAX3* [149], *RARG* [68], *RXYLT1* [33], *SEMA3A* [150], *SCLT1* [151], *SCRIB* [152], *SLC30A5* [139], *SNX3* [153], *TBX2* [135], *TBX3* [4], *TBX5* [135], *TLN1* [154], *TMEM98* [155], *ZIC2* [156], *ZNF219* [26], *ZNF503* [157], *ZNF703* [157]

† Syndromic and non-syndromic cases have been reported for some genes
‡ Only non-syndromic cases reported for all genes

References

1. Rivière, J. B.; Van Bon, B. W. M.; Hoischen, A.; Kholmanskikh, S. S.; O'Roak, B. J.; Gilissen, C.; Gijsen, S.; Sullivan, C. T.; Christian, S. L.; Abdul-Rahman, O. A.; et al. De Novo Mutations in the Actin Genes ACTB and ACTG1 Cause Baraitser-Winter Syndrome. *Nat. Genet.* **2012**, *44*, 440; DOI:10.1038/ng.1091
2. Rainger, J.; Williamson, K. A.; Soares, D. C.; Truch, J.; Kurian, D.; Gillessen-Kaesbach, G.; Seawright, A.; Prendergast, J.; Halachev, M.; Wheeler, A.; et al. A Recurrent de Novo Mutation in ACTG1 Causes Isolated Ocular Coloboma. *Hum. Mutat.* **2017**, *38*, 942; DOI:10.1002/humu.23246
3. Utsch, B.; Sayer, J. A.; Attanasio, M.; Pereira, R. R.; Eccles, M.; Hennies, H. C.; Otto, E. A.; & Hildebrandt, F. Identification of the First AHI1 Gene Mutations in Nephronophthisis-Associated Joubert Syndrome. *Pediatr. Nephrol.* **2006**, *21*, 32; DOI:10.1007/s00467-005-2054-y
4. ALSomiry, A. S.; Gregory-Evans, C. Y.; & Gregory-Evans, K. An Update on the Genetics of Ocular Coloboma. *Hum. Genet.* **2019**, *138*, 865; DOI:10.1007/s00439-019-02019-3
5. Uz, E.; Alanay, Y.; Aktas, D.; Vargel, I.; Gucer, S.; Tuncbilek, G.; von Eggeling, F.; Yilmaz, E.; Deren, O.; Posorski, N.; et al. Disruption of ALX1 Causes Extreme Microphthalmia and Severe Facial Clefting: Expanding the Spectrum of Autosomal-Recessive ALX-Related Frontonasal Dysplasia. *Am. J. Hum. Genet.* **2010**, *86*, 789; DOI:https://doi.org/10.1016/j.ajhg.2010.04.002
6. Twigg, S. R. F.; Versnel, S. L.; Nürnberg, G.; Lees, M. M.; Bhat, M.; Hammond, P.; Hennekam, R. C. M.; Hoogeboom, A. J. M.; Hurst, J. A.; Johnson, D.; et al. Frontorhiny, a Distinctive Presentation of Frontonasal Dysplasia Caused by Recessive Mutations in the ALX3 Homeobox Gene. *Am. J. Hum. Genet.* **2009**, *84*, 698; DOI:10.1016/j.ajhg.2009.04.009
7. Takagi, M.; Narumi, S.; Hamada, R.; Hasegawa, Y.; & Hasegawa, T. A Novel KAL1 Mutation Is Associated with Combined Pituitary Hormone Deficiency. *Hum. Genome Var.* **2014**, *1*, 14011; DOI:10.1038/hgv.2014.11
8. Shoubridge, C.; Jackson, M.; Grinton, B.; Berkovic, S. F.; Scheffer, I. E.; Huskins, S.; Thomas, A.; & Ware, T. Splice Variant in ARX Leading to Loss of C-Terminal Region in a Boy with Intellectual Disability and Infantile Onset Developmental and Epileptic Encephalopathy. *Am. J. Med. Genet. Part A* **2019**, *179*, 1483; DOI:10.1002/ajmg.a.61216
9. Stevens, E.; Carss, K. J.; Cirak, S.; Foley, A. R.; Torelli, S.; Willer, T.; Tambunan, D. E.; Yau, S.; Brodd, L.; Sewry, C. A.; et al. Mutations in B3GALNT2 Cause Congenital Muscular Dystrophy and Hypoglycosylation of α-Dystroglycan. *Am. J. Hum. Genet.* **2013**, *92*, 354; DOI:https://doi.org/10.1016/j.ajhg.2013.01.016
10. Reis, L. M. & Semina, E. V. *Conserved Genetic Pathways Associated with Microphthalmia, Anophthalmia, and Coloboma*, Birth Defects Research Part C - Embryo Today: Reviews; DOI:10.1002/bdrc.21097
11. Bakrania, P.; Efthymiou, M.; Klein, J. C.; Salt, A.; Bunyan, D. J.; Wyatt, A.; Ponting, C. P.; Martin, A.; Williams, S.; Lindley, V.; et al. Mutations in BMP4 Cause Eye, Brain, and Digit Developmental Anomalies: Overlap between the BMP4 and Hedgehog Signaling Pathways. *Am. J. Hum. Genet.* **2008**, *82*, 304; DOI:10.1016/j.ajhg.2007.09.023
12. Demeulenaere, S.; Beysen, D.; De Veuster, I.; Reyniers, E.; Kooy, F.; & Meuwissen, M. Novel BRPF1 Mutation in a Boy with Intellectual Disability, Coloboma, Facial Nerve Palsy and Hypoplasia of the Corpus Callosum. *Eur. J. Med. Genet.* **2019**, *62*, 5;

DOI:10.1016/j.ejmg.2019.103691
13. Hinds, A. M.; Rosser, E.; & Reddy, M. A. A Case of Exudative Vitreoretinopathy and Chorioretinal Coloboma Associated with Microcephaly in a Female with Contiguous Xp11.3-11.4 Deletion. *Ophthalmic Genet.* **2018**, *39*, 396; DOI:10.1080/13816810.2018.1443342
14. Kato, K.; Oka, Y.; Muramatsu, H.; Vasilev, F. F.; Otomo, T.; Oishi, H.; Kawano, Y.; Kidokoro, H.; Nakazawa, Y.; Ogi, T.; et al. Biallelic VPS35L Pathogenic Variants Cause 3C/Ritscher-Schinzel-like Syndrome through Dysfunction of Retriever Complex. *J. Med. Genet.* **2020**, *57*, 245 LP; DOI:10.1136/jmedgenet-2019-106213
15. Shaheen, R.; Patel, N.; Shamseldin, H.; Alzahrani, F.; Al-Yamany, R.; Almoisheer, A.; Ewida, N.; Anazi, S.; Alnemer, M.; Elsheikh, M.; et al. Accelerating Matchmaking of Novel Dysmorphology Syndromes through Clinical and Genomic Characterization of a Large Cohort. *Genet. Med.* **2016**, *18*, 686; DOI:10.1038/gim.2015.147
16. Berkun, L.; Slae, M.; Mor-Shaked, H.; Koplewitz, B.; Eventov-Friedman, S.; & Harel, T. Homozygous Variants in MAPRE2 and CDON in Individual with Skin Folds, Growth Delay, Retinal Coloboma, and Pyloric Stenosis. *Am. J. Med. Genet. Part A* **2019**, *179*, 2454; DOI:https://doi.org/10.1002/ajmg.a.61355
17. Reis, L. M.; Basel, D.; McCarrier, J.; Weinberg, D. V; & Semina, E. V Compound Heterozygous Splicing CDON Variants Result in Isolated Ocular Coloboma. *Clin. Genet.* **2020**, *98*, 486; DOI:https://doi.org/10.1111/cge.13824
18. Janssen, N.; Bergman, J. E. H.; Swertz, M. A.; Tranebjaerg, L.; Lodahl, M.; Schoots, J.; Hofstra, R. M. W.; Van Ravenswaaij-Arts, C. M. A.; & Hoefsloot, L. H. Mutation Update on the CHD7 Gene Involved in CHARGE Syndrome. *Hum. Mutat.* **2012**, *33*, 1149; DOI:10.1002/humu.22086
19. Deml, B.; Reis, L. M.; Lemyre, E.; Clark, R. D.; Kariminejad, A.; & Semina, E. V. Novel Mutations in PAX6, OTX2 and NDP in Anophthalmia, Microphthalmia and Coloboma. *Eur. J. Hum. Genet.* **2016**, *24*, 535; DOI:10.1038/ejhg.2015.155
20. Tonduti, D.; Pichiecchio, A.; La Piana, R.; Livingston, J. H.; Doherty, D. A.; Majumdar, A.; Tomkins, S.; Mine, M.; Ceroni, M.; Ricca, I.; et al. COL4A1-Related Disease: Raised Creatine Kinase and Cerebral Calcification as Useful Pointers. *Neuropediatrics* **2012**, *43*, 283; DOI:10.1055/s-0032-1325116
21. Indrieri, A.; Van Rahden, V. A.; Tiranti, V.; Morleo, M.; Iaconis, D.; Tammaro, R.; D'Amato, I.; Conte, I.; Maystadt, I.; Demuth, S.; et al. Mutations in COX7B Cause Microphthalmia with Linear Skin Lesions, an Unconventional Mitochondrial Disease. *Am. J. Hum. Genet.* **2012**, *91*, 942; DOI:10.1016/j.ajhg.2012.09.016
22. Beleggia, F.; Li, Y.; Fan, J.; Elcio lu, N. H.; Toker, E.; Wieland, T.; Maumenee, I. H.; Akarsu, N. A.; Meitinger, T.; Strom, T. M.; et al. CRIM1 Haploinsufficiency Causes Defects in Eye Development in Human and Mouse. *Hum. Mol. Genet.* **2015**, *24*, 2267; DOI:10.1093/hmg/ddu744
23. Shaheen, R.; Mark, P.; Prevost, C. T.; AlKindi, A.; Alhag, A.; Estwani, F.; Al-Sheddi, T.; Alobeid, E.; Alenazi, M. M.; Ewida, N.; et al. Biallelic Variants in CTU2 Cause DREAM-PL Syndrome and Impair Thiolation of TRNA Wobble U34. *Hum. Mutat.* **2019**, *40*, 2108; DOI:10.1002/humu.23870
24. Leibovitz, Z.; Mandel, H.; Falik-Zaccai, T. C.; Ben Harouch, S.; Savitzki, D.; Krajden-Haratz, K.; Gindes, L.; Tamarkin, M.; Lev, D.; Dobyns, W. B.; et al. Walker-Warburg Syndrome and Tectocerebellar Dysraphia: A Novel Association Caused by a Homozygous DAG1 Mutation. *Eur. J. Paediatr. Neurol.* **2018**, *22*, 525; DOI:https://doi.org/10.1016/j.ejpn.2017.12.012
25. Ajmal, M.; Khan, M. I.; Neveling, K.; Khan, Y. M.; Azam, M.; Waheed, N. K.; Hamel, C. P.; Ben-Yosef, T.; De Baere, E.; Koenekoop, R. K.; et al. A Missense Mutation in the Splicing Factor Gene DHX38 Is Associated with Early-Onset Retinitis Pigmentosa with Macular Coloboma. *J. Med. Genet.* **2014**, *51*, 444; DOI:10.1136/jmedgenet-2014-102316
26. Patel, N.; Khan, A. O.; Alsahli, S.; Abdel-Salam, G.; Nowilaty, S. R.; Mansour, A. M.; Nabil, A.; Al-Owain, M.; Sogati, S.; Salih, M. A.; et al. Genetic Investigation of 93 Families with Microphthalmia or Posterior Microphthalmos. *Clin. Genet.* **2018**, *93*, 1210; DOI:10.1111/cge.13239
27. Deml, B.; Reis, L. M.; Muheisen, S.; Bick, D.; & Semina, E. V. EFTUD2 Deficiency in Vertebrates: Identification of a Novel Human Mutation and Generation of a Zebrafish Model. *Birth Defects Res. Part A - Clin. Mol. Teratol.* **2015**, *103*, 630; DOI:10.1002/bdra.23397
28. Masuda, K.; Akiyama, K.; Arakawa, M.; Nishi, E.; Kitazawa, N.; Higuchi, T.; Katou, Y.; Shirahige, K.; & Izumi, K. Exome Sequencing Identification of EP300 Mutation in a Proband with Coloboma and

Imperforate Anus: Possible Expansion of the Phenotypic Spectrum of Rubinstein-Taybi Syndrome. *Mol. Syndromol.* **2015**, *6*, 99; DOI:10.1159/000375542
29. Jaspers, N. G. J.; Raams, A.; Silengo, M. C.; Wijgers, N.; Niedernhofer, L. J.; Robinson, A. R.; Giglia-Mari, G.; Hoogstraten, D.; Kleijer, W. J.; Hoeijmakers, J. H. J.; et al. First Reported Patient with Human ERCC1 Deficiency Has Cerebro-Oculo-Facio-Skeletal Syndrome with a Mild Defect in Nucleotide Excision Repair and Severe Developmental Failure. *Am. J. Hum. Genet.* **2007**, *80*, 457; DOI:10.1086/512486
30. Drury, S.; Boustred, C.; Tekman, M.; Stanescu, H.; Kleta, R.; Lench, N.; Chitty, L. S.; & Scott, R. H. A Novel Homozygous ERCC5 Truncating Mutation in a Family with Prenatal Arthrogryposis—Further Evidence of Genotype–Phenotype Correlation. *Am. J. Med. Genet. Part A* **2014**, *164*, 1777; DOI:10.1002/ajmg.a.36506
31. Jaakkola, E.; Mustonen, A.; Olsen, P.; Miettinen, S.; Savuoja, T.; Raams, A.; Jaspers, N. G. J.; Shao, H.; Wu, B. L.; & Ignatius, J. ERCC6 Founder Mutation Identified in Finnish Patients with COFS Syndrome. *Clin. Genet.* **2010**, *78*, 541; DOI:10.1111/j.1399-0004.2010.01424.x
32. Unger, S.; Górna, M. W.; Le Béchec, A.; Do Vale-Pereira, S.; Bedeschi, M. F.; Geiberger, S.; Grigelioniene, G.; Horemuzova, E.; Lalatta, F.; Lausch, E.; et al. FAM111A Mutations Result in Hypoparathyroidism and Impaired Skeletal Development. *Am. J. Hum. Genet.* **2013**, *92*, 990; DOI:https://doi.org/10.1016/j.ajhg.2013.04.020
33. Eintracht, J.; Corton, M.; FitzPatrick, D.; & Moosajee, M. CUGC for Syndromic Microphthalmia Including Next-Generation Sequencing-Based Approaches. *Eur. J. Hum. Genet.* **2020**,; DOI:10.1038/s41431-019-0565-4
34. Lahrouchi, N.; George, A.; Ratbi, I.; Schneider, R.; Elalaoui, S. C.; Moosa, S.; Bharti, S.; Sharma, R.; Abu-Asab, M.; Onojafe, F.; et al. Homozygous Frameshift Mutations in FAT1 Cause a Syndrome Characterized by Colobomatous-Microphthalmia, Ptosis, Nephropathy and Syndactyly. *Nat. Commun.* **2019**, *10*, 1; DOI:10.1038/s41467-019-08547-w
35. Holt, R. J.; Young, R. M.; Crespo, B.; Ceroni, F.; Curry, C. J.; Bellacchio, E.; Bax, D. A.; Ciolfi, A.; Simon, M.; Fagerberg, C. R.; et al. De Novo Missense Variants in FBXW11 Cause Diverse Developmental Phenotypes Including Brain, Eye, and Digit Anomalies. *Am. J. Hum. Genet.* **2019**, *105*, 640; DOI:https://doi.org/10.1016/j.ajhg.2019.07.005
36. Limoge, F.; Picot, D.; Masurel, A.; Terriat, B.; Champilou, C.; & Minot, D. Clinical Exome | Genome Reports Homozygous FIBP Nonsense Variant Responsible of Syndromic Overgrowth , with Overgrowth , Macrocephaly , Retinal Coloboma. **2015**, 1
37. Beltran-Valero de Bernabé, D.; Voit, T.; Longman, C.; Steinbrecher, A.; Straub, V.; Yuva, Y.; Herrmann, R.; Sperner, J.; Korenke, C.; Diesen, C.; et al. Mutations in the FKRP Gene Can Cause Muscle-Eye-Brain Disease and Walker-Warburg Syndrome. *J. Med. Genet.* **2004**, *41*, 1; DOI:10.1136/jmg.2003.013870
38. Kondo, Y.; Koshimizu, E.; Megarbane, A.; Hamanoue, H.; Okada, I.; Nishiyama, K.; Kodera, H.; Miyatake, S.; Tsurusaki, Y.; Nakashima, M.; et al. Whole-Exome Sequencing Identified a Homozygous FNBP4 Mutation in a Family with a Condition Similar to Microphthalmia with Limb Anomalies. *Am. J. Med. Genet. Part A* **2013**, *161*, 1543; DOI:https://doi.org/10.1002/ajmg.a.35983
39. Giri, D.; Vignola, M. L.; Gualtieri, A.; Scagliotti, V.; McNamara, P.; Peak, M.; Didi, M.; Gaston-Massuet, C.; & Senniappan, S. Novel FOXA2 Mutation Causes Hyperinsulinism, Hypopituitarism with Craniofacial and Endoderm-Derived Organ Abnormalities. *Hum. Mol. Genet.* **2017**, *26*, 4315; DOI:10.1093/hmg/ddx318
40. Zhang, X.; Wang, D.; Dongye, M.; Zhu, Y.; Chen, C.; Wang, R.; Long, E.; Liu, Z.; Wu, X.; Lin, D.; et al. Loss-of-Function Mutations in FREM2 Disrupt Eye Morphogenesis. *Exp. Eye Res.* **2019**, *181*, 302; DOI:https://doi.org/10.1016/j.exer.2019.02.013
41. Vitiello, C.; D'Adamo, P.; Gentile, F.; Vingolo, E. M.; Gasparini, P.; & Banfi, S. A Novel GJA1 Mutation Causes Oculodentodigital Dysplasia without Syndactyly. *Am. J. Med. Genet. Part A* **2005**, *133A*, 58; DOI:10.1002/ajmg.a.30554
42. Patel, N.; Shamseldin, H. E.; Sakati, N.; Khan, A. O.; Softa, A.; Al-Fadhli, F. M.; Hashem, M.; Abdulwahab, F. M.; Alshidi, T.; Alomar, R.; et al. GZF1 Mutations Expand the Genetic Heterogeneity of Larsen Syndrome. *Am. J. Hum. Genet.* **2017**, *100*, 831; DOI:10.1016/j.ajhg.2017.04.008
43. Sobrier, M.-L.; Netchine, I.; Heinrichs, C.; Thibaud, N.; Vié-Luton, M.-P.; Van Vliet, G.; & Amselem, S. Alu-Element Insertion in the Homeodomain of HESX1 and Aplasia of the Anterior Pituitary. *Hum.*

Mutat. **2005**, *25*, 503; DOI:https://doi.org/10.1002/humu.9332
44. Bardakjian, T.; Weiss, A.; & Schneider, A. Microphthalmia / Anophthalmia / Coloboma Spectrum Summary Clinical Characteristics Genetic Counseling. **2020**, 1
45. Wang, S. F.; Kowal, T. J.; Ning, K.; Koo, E. B.; Wu, A. Y.; Mahajan, V. B.; & Sun, Y. Review of Ocular Manifestations of Joubert Syndrome. *Genes (Basel).* **2018**, *9*; DOI:10.3390/genes9120605
46. Krall, M.; Htun, S.; Schnur, R. E.; Brooks, A. S.; Baker, L.; de Alba Campomanes, A.; Lamont, R. E.; Gripp, K. W.; Schneidman-Duhovny, D.; Innes, A. M.; et al. Biallelic Sequence Variants in INTS1 in Patients with Developmental Delays, Cataracts, and Craniofacial Anomalies. *Eur. J. Hum. Genet.* **2019**, *27*, 582; DOI:10.1038/s41431-018-0298-9
47. Gueneau, L.; Fish, R. J.; Shamseldin, H. E.; Voisin, N.; Tran Mau-Them, F.; Preiksaitiene, E.; Monroe, G. R.; Lai, A.; Putoux, A.; Allias, F.; et al. KIAA1109 Variants Are Associated with a Severe Disorder of Brain Development and Arthrogryposis. *Am. J. Hum. Genet.* **2018**, *102*, 116; DOI:https://doi.org/10.1016/j.ajhg.2017.12.002
48. Dafsari, H. S.; Byrne, S.; Lin, J.-P.; Pitt, M.; Jongbloed, J. D. H.; Flinter, F.; & Jungbluth, H. Goldberg–Shprintzen Megacolon Syndrome with Associated Sensory Motor Axonal Neuropathy. *Am. J. Med. Genet. Part A* **2015**, *167*, 1300; DOI:https://doi.org/10.1002/ajmg.a.36873
49. Jones, G. E.; Ostergaard, P.; Moore, A. T.; Connell, F. C.; Williams, D.; Quarrell, O.; Brady, A. F.; Spier, I.; Hazan, F.; Moldovan, O.; et al. Microcephaly with or without Chorioretinopathy, Lymphoedema, or Mental Retardation (MCLMR): Review of Phenotype Associated with KIF11 Mutations. *Eur. J. Hum. Genet.* **2014**, *22*, 881; DOI:10.1038/ejhg.2013.263
50. Okumura, T.; Furuichi, K.; Higashide, T.; Sakurai, M.; Hashimoto, S.; Shinozaki, Y.; Hara, A.; Iwata, Y.; Sakai, N.; Sugiyama, K.; et al. Association of PAX2 and Other Gene Mutations with the Clinical Manifestations of Renal Coloboma Syndrome. *PLoS One* **2015**, *10*, 1; DOI:10.1371/journal.pone.0142843
51. Badalato, L.; Farhan, S. M. K.; Dilliott, A. A.; Bulman, D. E.; Hegele, R. A.; & Goobie, S. L. KMT2D p.Gln3575His Segregating in a Family with Autosomal Dominant Choanal Atresia Strengthens the Kabuki/CHARGE Connection. *Am. J. Med. Genet. Part A* **2017**, *173*, 183; DOI:10.1002/ajmg.a.38010
52. Giordano, M.; Muratore, V.; Babu, D.; Meazza, C.; & Bozzola, M. A 18p11.23-P11.31 Microduplication in a Boy with Psychomotor Delay, Cerebellar Vermis Hypoplasia, Chorioretinal Coloboma, Deafness and GH Deficiency. *Mol. Cytogenet.* **2016**, *9*, 10; DOI:10.1186/s13039-016-0298-9
53. Isrie, M.; Breuss, M.; Tian, G.; Hansen, A. H.; Cristofoli, F.; Morandell, J.; Kupchinsky, Z. A.; Sifrim, A.; Rodriguez-Rodriguez, C. M.; Dapena, E. P.; et al. Mutations in Either TUBB or MAPRE2 Cause Circumferential Skin Creases Kunze Type. *Am. J. Hum. Genet.* **2015**, *97*, 790; DOI:https://doi.org/10.1016/j.ajhg.2015.10.014
54. Nizon, M.; Laugel, V.; Flanigan, K. M.; Pastore, M.; Waldrop, M. A.; Rosenfeld, J. A.; Marom, R.; Xiao, R.; Gerard, A.; Pichon, O.; et al. Variants in MED12L, Encoding a Subunit of the Mediator Kinase Module, Are Responsible for Intellectual Disability Associated with Transcriptional Defect. *Genet. Med.* **2019**, *21*, 2713; DOI:10.1038/s41436-019-0557-3
55. Cavodeassi, F.; Creuzet, S.; & Etchevers, H. C. The Hedgehog Pathway and Ocular Developmental Anomalies. *Hum. Genet.* **2019**, *138*, 917; DOI:10.1007/s00439-018-1918-8
56. Mohamed, F. E.; Al Sorkhy, M.; Ghattas, M. A.; Al-Zaabi, N.; Al-Shamsi, A.; Almansoori, T. M.; Al-Gazali, L.; Al-Dirbashi, O. Y.; Al-Jasmi, F.; & Ali, B. R. A Novel Homozygous Missense Variant in the NAGA Gene with Extreme Intrafamilial Phenotypic Heterogeneity. *J. Mol. Neurosci.* **2020**, *70*, 45; DOI:10.1007/s12031-019-01398-6
57. van Rahden, V. A.; Fernandez-Vizarra, E.; Alawi, M.; Brand, K.; Fellmann, F.; Horn, D.; Zeviani, M.; & Kutsche, K. Mutations in NDUFB11, Encoding a Complex I Component of the Mitochondrial Respiratory Chain, Cause Microphthalmia with Linear Skin Defects Syndrome. *Am. J. Hum. Genet.* **2015**, *96*, 640; DOI:10.1016/j.ajhg.2015.02.002
58. Wu, B. L.; Austin, M. A.; Schneider, G. H.; Boles, R. G.; & Korf, B. R. Deletion of the Entire NF1 Gene Detected by Fish: Four Deletion Patients Associated with Severe Manifestations. *Am. J. Med. Genet.* **1995**, *59*, 528; DOI:10.1002/ajmg.1320590427
59. Bisgaard, A. M.; Rasmussen, L. N.; Møller, H. U.; Kirchhoff, M.; & Bryndorf, T. Interstitial Deletion of the Short Arm of Chromosome 1 (1p13.1p21.1) in a Girl with Mental Retardation, Short Stature and Colobomata. *Clin. Dysmorphol.* **2007**, *16*, 109; DOI:10.1097/01.mcd.0000228425.89660.bf

60. Yang, Y.; He, Y.; Zhen, L.; & Li, D.-Z. Fetal Phenotype of Galloway-Mowat Syndrome 3 Caused by a Specific <em>OSGEP</Em> Variant. *Eur. J. Obstet. Gynecol. Reprod. Biol.* **2019**, *242*, 182; DOI:10.1016/j.ejogrb.2019.09.025
61. Thomas, S.; Wright, K. J.; Corre, S. Le; Micalizzi, A.; Romani, M.; Abhyankar, A.; Saada, J.; Perrault, I.; Amiel, J.; Litzler, J.; et al. A Homozygous PDE6D Mutation in Joubert Syndrome Impairs Targeting of Farnesylated INPP5E Protein to the Primary Cilium. *Hum. Mutat.* **2014**, *35*, 137; DOI:10.1002/humu.22470
62. Ng, B. G.; Hackmann, K.; Jones, M. A.; Eroshkin, A. M.; He, P.; Wiliams, R.; Bhide, S.; Cantagrel, V.; Gleeson, J. G.; Paller, A. S.; et al. Mutations in the Glycosylphosphatidylinositol Gene PIGL Cause CHIME Syndrome. *Am. J. Hum. Genet.* **2012**, *90*, 685; DOI:10.1016/j.ajhg.2012.02.010
63. Hanemaaijer, N.; Dijkhuizen, T.; Haadsma, M.; Boeve, M.; Boon, M.; Hordijk, R.; Kok, K.; Sikkema-Raddatz, B.; & van Ravenswaaij-Arts, C. M. A. A 649 Kb Microduplication in 1p34.1, Including POMGNT1, in a Patient with Microcephaly, Coloboma and Laryngomalacia; and a Review of the Literature. *Eur. J. Med. Genet.* **2009**, *52*, 116; DOI:10.1016/j.ejmg.2009.01.005
64. Rupprich, K.; Kölbel, H.; Sánchez-Albisua, I.; Marina, A. Della; Stein, A.; Küchler, A.; Elgizouli, M.; Schweiger, B.; Hehr, U.; & Schara, U. P 307. Walker–Warburg's Syndrome Due to Homozygous POMK Mutation in Preterm Twins: Case Report. *Neuropediatrics* **2018**, *49*, P307; DOI:10.1055/s-0038-1675963
65. Vajsar, J.; Baskin, B.; Swoboda, K.; Biggar, D. W.; Schachter, H.; & Ray, P. N. Walker-Warburg Syndrome with POMT1 Mutations Can Be Associated with Cleft Lip and Cleft Palate. *Neuromuscul. Disord.* **2008**, *18*, 675; DOI:https://doi.org/10.1016/j.nmd.2008.05.014
66. Low, K. J.; Ansari, M.; Abou Jamra, R.; Clarke, A.; El Chehadeh, S.; Fitzpatrick, D. R.; Greenslade, M.; Henderson, A.; Hurst, J.; Keller, K.; et al. PUF60 Variants Cause a Syndrome of ID, Short Stature, Microcephaly, Coloboma, Craniofacial, Cardiac, Renal and Spinal Features. *Eur. J. Hum. Genet.* **2017**, *25*, 552; DOI:10.1038/ejhg.2017.27
67. Bem, D.; Yoshimura, S. I.; Nunes-Bastos, R.; Bond, F. F.; Kurian, M. A.; Rahman, F.; Handley, M. T. W.; Hadzhiev, Y.; Masood, I.; Straatman-Iwanowska, A. A.; et al. Loss-of-Function Mutations in RAB18 Cause Warburg Micro Syndrome. *Am. J. Hum. Genet.* **2011**, *88*, 499; DOI:10.1016/j.ajhg.2011.03.012
68. Jakubiuk-Tomaszuk, A.; Murcia Pienkowski, V.; Zietkiewicz, S.; Rydzanicz, M.; Kosińska, J.; Stawiński, P.; Szumiński, M.; & Płoski, R. Syndromic Chorioretinal Coloboma Associated with Heterozygous de Novo RARA Mutation Affecting an Amino Acid Critical for Retinoic Acid Interaction. *Clin. Genet.* **2019**, *96*, 371; DOI:10.1111/cge.13611
69. Fregeau, B.; Kim, B. J.; Hernández-García, A.; Jordan, V. K.; Cho, M. T.; Schnur, R. E.; Monaghan, K. G.; Juusola, J.; Rosenfeld, J. A.; Bhoj, E.; et al. De Novo Mutations of RERE Cause a Genetic Syndrome with Features That Overlap Those Associated with Proximal 1p36 Deletions. *Am. J. Hum. Genet.* **2016**, *98*, 963; DOI:10.1016/j.ajhg.2016.03.002
70. Kalay, E.; Sezgin, O.; Chellappa, V.; Mutlu, M.; Morsy, H.; Kayserili, H.; Kreiger, E.; Cansu, A.; Toraman, B.; Abdalla, E. M.; et al. Mutations in RIPK4 Cause the Autosomal-Recessive Form of Popliteal Pterygium Syndrome. *Am. J. Hum. Genet.* **2012**, *90*, 76; DOI:https://doi.org/10.1016/j.ajhg.2011.11.014
71. Doherty, D.; Parisi, M. A.; Finn, L. S.; Gunay-Aygun, M.; Al-Mateen, M.; Bates, D.; Clericuzio, C.; Demir, H.; Dorschner, M.; Van Essen, A. J.; et al. Mutations in 3 Genes (MKS3, CC2D2A and RPGRIP1L) Cause COACH Syndrome (Joubert Syndrome with Congenital Hepatic Fibrosis). *J. Med. Genet.* **2010**, *47*, 8; DOI:10.1136/jmg.2009.067249
72. Lalani, S. R.; Safiullah, A. M.; Molinari, L. M.; Fernbach, S. D.; Martin, D. M.; & Belmont, J. W. SEMA3E Mutation in a Patient with CHARGE Syndrome. *J. Med. Genet.* **2004**, *41*, 2; DOI:10.1136/jmg.2003.017640
73. Chang, T. C.; Bauer, M.; Puerta, H. S.; Greenberg, M. B.; & Cavuoto, K. M. Ophthalmic Findings in Frank-Ter Haar Syndrome: Report of a Sibling Pair. *J. AAPOS* **2017**, *21*, 514; DOI:10.1016/j.jaapos.2017.07.216
74. Ehmke, N.; Graul-Neumann, L.; Smorag, L.; Koenig, R.; Segebrecht, L.; Magoulas, P.; Scaglia, F.; Kilic, E.; Hennig, A. F.; Adolphs, N.; et al. De Novo Mutations in SLC25A24 Cause a Craniosynostosis Syndrome with Hypertrichosis, Progeroid Appearance, and Mitochondrial Dysfunction. *Am. J. Hum. Genet.* **2017**, *101*, 833; DOI:https://doi.org/10.1016/j.ajhg.2017.09.016
75. Poulter, J. A.; Al-Araimi, M.; Conte, I.; van Genderen, M. M.; Sheridan, E.; Carr, I. M.; Parry, D. A.; Shires, M.; Carrella, S.;

Bradbury, J.; et al. Recessive Mutations in SLC38A8 Cause Foveal Hypoplasia and Optic Nerve Misrouting without Albinism. *Am. J. Hum. Genet.* **2013**, *93*, 1143; DOI:https://doi.org/10.1016/j.ajhg.2013.11.002
76. Errichiello, E.; Mustafa, N.; Vetro, A.; Notarangelo, L. D.; de Jonge, H.; Rinaldi, B.; Vergani, D.; Giglio, S. R.; Morbini, P.; & Zuffardi, O. SMARCA4 Inactivating Mutations Cause Concomitant Coffin–Siris Syndrome, Microphthalmia and Small-Cell Carcinoma of the Ovary Hypercalcaemic Type. *J. Pathol.* **2017**, *243*, 9; DOI:10.1002/path.4926
77. Lecoquierre, F.; Bonnevalle, A.; Chadie, A.; Gayet, C.; Dumant-Forest, C.; Renaux-Petel, M.; Leca, J.-B.; Hazelzet, T.; Brasseur-Daudruy, M.; Louillet, F.; et al. Confirmation and Further Delineation of the SMG9-deficiency Syndrome, a Rare and Severe Developmental Disorder. *Am. J. Med. Genet. Part A* **2019**, *179*, 2257; DOI:10.1002/ajmg.a.61317
78. Twigg, S. R. F.; Hufnagel, R. B.; Miller, K. A.; Zhou, Y.; McGowan, S. J.; Taylor, J.; Craft, J.; Taylor, J. C.; Santoro, S. L.; Huang, T.; et al. A Recurrent Mosaic Mutation in SMO , Encoding the Hedgehog Signal Transducer Smoothened, Is the Major Cause of Curry-Jones Syndrome. *Am. J. Hum. Genet.* **2016**, *98*, 1256; DOI:10.1016/j.ajhg.2016.04.007
79. Jelsig, A. M.; Diness, B. R.; Kreiborg, S.; Main, K. M.; Larsen, V. A.; & Hove, H. A Complex Phenotype in a Family with a Pathogenic SOX3 Missense Variant. *Eur. J. Med. Genet.* **2018**, *61*, 168; DOI:10.1016/j.ejmg.2017.11.012
80. Bondurand, N.; Kuhlbrodt, K.; Pingault, V.; Enderich, J.; Sajus, M.; Tommerup, N.; Warburg, M.; Hennekam, R. C. M.; Read, A. P.; Wegner, M.; et al. A Molecular Analysis of the Yemenite Deaf-Blind Hypopigmentation Syndrome: SOX10 Dysfunction Causes Different Neurocristopathies. *Hum. Mol. Genet.* **1999**, *8*, 1785; DOI:10.1093/hmg/8.9.1785
81. Hirabayashi, K. E.; Moore, A. T.; Mendelsohn, B. A.; Taft, R. J.; Chawla, A.; Perry, D.; Henry, D.; & Slavotinek, A. Congenital Sodium Diarrhea and Chorioretinal Coloboma with Optic Disc Coloboma in a Patient with Biallelic SPINT2 Mutations, Including p.(Tyr163Cys). *Am. J. Med. Genet. Part A* **2018**, *176*, 997; DOI:10.1002/ajmg.a.38637
82. Kara, B.; Ayhan, Ö.; Gökçay, G.; Başboğaoğlu, N.; & Tolun, A. Adult Phenotype and Further Phenotypic Variability in SRD5A3-CDG. *BMC Med. Genet.* **2014**, *15*; DOI:10.1186/1471-2350-15-10
83. Liegel, R. P.; Handley, M. T.; Ronchetti, A.; Brown, S.; Langemeyer, L.; Linford, A.; Chang, B.; Morris-Rosendahl, D. J.; Carpanini, S.; Posmyk, R.; et al. Loss-of-Function Mutations in TBC1D20 Cause Cataracts and Male Infertility in Blind Sterile Mice and Warburg Micro Syndrome in Humans. *Am. J. Hum. Genet.* **2013**, *93*, 1001; DOI:10.1016/j.ajhg.2013.10.011
84. Pauws, E.; Peskett, E.; Boissin, C.; Hoshino, A.; Mengrelis, K.; Carta, E.; Abruzzo, M. A.; Lees, M.; Moore, G. E.; Erickson, R. P.; et al. X-Linked CHARGE-like Abruzzo-Erickson Syndrome and Classic Cleft Palate with Ankyloglossia Result from TBX22 Splicing Mutations. *Clin. Genet.* **2013**, *83*, 352; DOI:10.1111/j.1399-0004.2012.01930.x
85. Gestri, G.; Osborne, R. J.; Wyatt, A. W.; Gerrelli, D.; Gribble, S.; Stewart, H.; Fryer, A.; Bunyan, D. J.; Prescott, K.; Collin, J. R. O.; et al. Reduced TFAP2A Function Causes Variable Optic Fissure Closure and Retinal Defects and Sensitizes Eye Development to Mutations in Other Morphogenetic Regulators. *Hum. Genet.* **2009**, *126*, 791; DOI:10.1007/s00439-009-0730-x
86. Brancati, F.; Iannicelli, M.; Travaglini, L.; Mazzotta, A.; Bertini, E.; Boltshauser, E.; D'Arrigo, S.; Emma, F.; Fazzi, E.; Gallizzi, R.; et al. MKS3/TMEM67 Mutations Are a Major Cause of COACH Syndrome, a Joubert Syndrome Related Disorder with Liver Involvement. *Hum. Mutat.* **2009**, *30*, E432; DOI:10.1002/humu.20924
87. Edvardson, S.; Shaag, A.; Zenvirt, S.; Erlich, Y.; Hannon, G. J.; Shanske, A. L.; Gomori, J. M.; Ekstein, J.; & Elpeleg, O. Joubert Syndrome 2 (JBTS2) in Ashkenazi Jews Is Associated with a TMEM216 Mutation. *Am. J. Hum. Genet.* **2010**, *86*, 93; DOI:https://doi.org/10.1016/j.ajhg.2009.12.007
88. Myers, K. A.; Bello-Espinosa, L. E.; Kherani, A.; Wei, X.-C.; & Innes, A. M. TUBA1A Mutation Associated With Eye Abnormalities in Addition to Brain Malformation. *Pediatr. Neurol.* **2015**, *53*, 442; DOI:https://doi.org/10.1016/j.pediatrneurol.2015.07.004
89. Scheidecker, S.; Etard, C.; Haren, L.; Stoetzel, C.; Hull, S.; Arno, G.; Plagnol, V.; Drunat, S.; Passemard, S.; Toutain, A.; et al. Mutations in TUBGCP4 Alter Microtubule Organization via the γ-Tubulin Ring Complex in Autosomal-Recessive Microcephaly with Chorioretinopathy. *Am. J. Hum. Genet.* **2015**, *96*, 666; DOI:https://doi.org/10.1016/j.ajhg.2015.02.011
90. Slavotinek, A. M.; Chao, R.; Vacik, T.; Yahyavi, M.; Abouzeid, H.;

Bardakjian, T.; Schneider, A.; Shaw, G.; Sherr, E. H.; Lemke, G.; et al. VAX1 Mutation Associated with Microphthalmia, Corpus Callosum Agenesis, and Orofacial Clefting: The First Description of a VAX1 Phenotype in Humans. *Hum. Mutat.* **2012**, *33*, 364; DOI:https://doi.org/10.1002/humu.21658

91. Elliott, A. M.; Simard, L. R.; Coghlan, G.; Chudley, A. E.; Chodirker, B. N.; Greenberg, C. R.; Burch, T.; Ly, V.; Hatch, G. M.; & Zelinski, T. A Novel Mutation in KIAA0196: Identification of a Gene Involved in Ritscher-Schinzel/3C Syndrome in a First Nations Cohort. *J. Med. Genet.* **2013**, *50*, 819; DOI:10.1136/jmedgenet-2013-101715
92. Sutani, A.; Shima, H.; Hijikata, A.; Hosokawa, S.; Katoh-Fukui, Y.; Takasawa, K.; Suzuki, E.; Doi, S.; Shirai, T.; Morio, T.; et al. WDR11 Is Another Causative Gene for Coloboma, Cardiac Anomaly and Growth Retardation in 10q26 Deletion Syndrome. *Eur. J. Med. Genet.* **2020**, *63*, 103626; DOI:https://doi.org/10.1016/j.ejmg.2019.01.016
93. Kanca, O.; Andrews, J. C.; Lee, P.-T.; Patel, C.; Braddock, S. R.; Slavotinek, A. M.; Cohen, J. S.; Gubbels, C. S.; Aldinger, K. A.; Williams, J.; et al. De Novo Variants in WDR37 Are Associated with Epilepsy, Colobomas, Dysmorphism, Developmental Delay, Intellectual Disability, and Cerebellar Hypoplasia. *Am. J. Hum. Genet.* **2019**, *105*, 413; DOI:https://doi.org/10.1016/j.ajhg.2019.06.014
94. Williamson, K. A. & FitzPatrick, D. R. The Genetic Architecture of Microphthalmia, Anophthalmia and Coloboma. *Eur. J. Med. Genet.* **2014**, *57*, 369; DOI:10.1016/j.ejmg.2014.05.002
95. Gregory-Evans, C. Y.; Vieira, H.; Dalton, R.; Adams, G. G. W.; Salt, A.; & Gregory-Evans, K. Ocular Coloboma and High Myopia with Hirschsprung Disease Associated with a Novel ZFHX1B Missense Mutation and Trisomy 21. *Am. J. Med. Genet.* **2004**, *131A*, 86; DOI:10.1002/ajmg.a.30312
96. Carapito, R.; Ivanova, E. L.; Morlon, A.; Meng, L.; Molitor, A.; Erdmann, E.; Kieffer, B.; Pichot, A.; Naegely, L.; Kolmer, A.; et al. ZMIZ1 Variants Cause a Syndromic Neurodevelopmental Disorder. *Am. J. Hum. Genet.* **2019**, *104*, 319; DOI:https://doi.org/10.1016/j.ajhg.2018.12.007
97. Wang, L.; He, F.; Bu, J.; Liu, X.; Du, W.; Dong, J.; Cooney, J. D.; Dubey, S. K.; Shi, Y.; Gong, B.; et al. ABCB6 Mutations Cause Ocular Coloboma. *Am. J. Hum. Genet.* **2012**, *90*, 40; DOI:10.1016/j.ajhg.2011.11.026
98. Beby, F.; Commeaux, C.; Bozon, M.; Denis, P.; Edery, P.; & Morlé, L. New Phenotype Associated with an Arg116Cys Mutation in the CRYAA Gene: Nuclear Cataract, Iris Coloboma, and Microphthalmia. *Arch. Ophthalmol.* **2007**, *125*, 213; DOI:10.1001/archopht.125.2.213
99. Ceroni, F.; Aguilera-Garcia, D.; Chassaing, N.; Bax, D. A.; Blanco-Kelly, F.; Ramos, P.; Tarilonte, M.; Villaverde, C.; da Silva, L. R. J.; Ballesta-Martínez, M. J.; et al. New GJA8 Variants and Phenotypes Highlight Its Critical Role in a Broad Spectrum of Eye Anomalies. *Hum. Genet.* **2019**, *138*, 1027; DOI:10.1007/s00439-018-1875-2
100. Vidya, N. G.; Rajkumar, S.; & Vasavada, A. R. Genetic Investigation of Ocular Developmental Genes in 52 Patients with Anophthalmia/Microphthalmia. *Ophthalmic Genet.* **2018**, *39*, 344; DOI:10.1080/13816810.2018.1436184
101. Huang, X.-F.; Xiang, L.; Cheng, W.; Cheng, F.-F.; He, K.-W.; Zhang, B.-W.; Zheng, S.-S.; Han, R.-Y.; Zheng, Y.-H.; Xu, X.-T.; et al. Mutation of IPO13 Causes Recessive Ocular Coloboma, Microphthalmia, and Cataract. *Exp. Mol. Med.* **2018**, *50*, 53; DOI:10.1038/s12276-018-0079-0
102. Jamieson, R. V; Perveen, R.; Kerr, B.; Carette, M.; Yardley, J.; Heon, E.; Wirth, M. G.; van Heyningen, V.; Donnai, D.; Munier, F.; et al. Domain Disruption and Mutation of the BZIP Transcription Factor, MAF,Associated with Cataract, Ocular Anterior Segment Dysgenesis and Coloboma. *Hum. Mol. Genet.* **2002**, *11*, 33; DOI:10.1093/hmg/11.1.33
103. Holt, R.; Ugur Iseri, S. A.; Wyatt, A. W.; Bax, D. A.; Gold Diaz, D.; Santos, C.; Broadgate, S.; Dunn, R.; Bruty, J.; Wallis, Y.; et al. Identification and Functional Characterisation of Genetic Variants in OLFM2 in Children with Developmental Eye Disorders. *Hum. Genet.* **2017**, *136*, 119; DOI:10.1007/s00439-016-1745-8
104. Matías-Pérez, D.; García-Montaño, L. A.; Cruz-Aguilar, M.; García-Montalvo, I. A.; Nava-Valdéz, J.; Barragán-Arevalo, T.; Villanueva-Mendoza, C.; Villarroel, C. E.; Guadarrama-Vallejo, C.; la Cruz, R. V.; et al. Identification of Novel Pathogenic Variants and Novel Gene-Phenotype Correlations in Mexican Subjects with Microphthalmia and/or Anophthalmia by next-Generation Sequencing. *J. Hum. Genet.* **2018**, *63*, 1169; DOI:10.1038/s10038-018-0504-1
105. Chou, C. M.; Nelson, C.; Tarlé, S. A.; Pribila, J. T.; Bardakjian, T.; Woods, S.; Schneider, A.; & Glaser, T. Biochemical Basis for Dominant Inheritance, Variable Penetrance, and Maternal Effects in

RBP4 Congenital Eye Disease. *Cell* **2015**, *161*, 634; DOI:10.1016/j.cell.2015.03.006
106. Cukras, C.; Gaasterland, T.; Lee, P.; Gudiseva, H. V.; Chavali, V. R. M.; Pullakhandam, R.; Maranhao, B.; Edsall, L.; Soares, S.; Reddy, G. B.; et al. Exome Analysis Identified a Novel Mutation in the RBP4 Gene in a Consanguineous Pedigree with Retinal Dystrophy and Developmental Abnormalities. *PLoS One* **2012**, *7*; DOI:10.1371/journal.pone.0050205
107. Chassaing, N.; Ragge, N.; Plaisancié, J.; Patat, O.; Geneviève, D.; Rivier, F.; Malrieu-Eliaou, C.; Hamel, C.; Kaplan, J.; & Calvas, P. Confirmation of TENM3 Involvement in Autosomal Recessive Colobomatous Microphthalmia. *Am. J. Med. Genet. Part A* **2016**, *170*, 1895; DOI:https://doi.org/10.1002/ajmg.a.37667
108. Chao, R.; Nevin, L.; Agarwal, P.; Riemer, J.; Bai, X.; Delaney, A.; Akana, M.; JimenezLopez, N.; Bardakjian, T.; Schneider, A.; et al. A Male with Unilateral Microphthalmia Reveals a Role for TMX3 in Eye Development. *PLoS One* **2010**, *5*, e10565; DOI:10.1371/journal.pone.0010565
109. Cao, M.; Ouyang, J.; Guo, J.; Lin, S.; & Chen, S. Metalloproteinase Adamts16 Is Required for Proper Closure of the Optic Fissure. *Invest. Ophthalmol. Vis. Sci.* **2018**, *59*, 1167; DOI:10.1167/iovs.17-22827
110. Babcock, H. E.; Dutta, S.; Alur, R. P.; Brocker, C.; Vasiliou, V.; Vitale, S.; Abu-Asab, M.; & Brooks, B. P. Aldh7a1 Regulates Eye and Limb Development in Zebrafish. *PLoS One* **2014**, *9*, e101782
111. Lee, J. & Gross, J. M. Function and Regulation of Bcl6 During Zebrafish Eye Development. *Invest. Ophthalmol. Vis. Sci.* **2010**, *51*, 726
112. Anjanappa, R. M.; Nayak, S.; Moily, N. S.; Manduva, V.; Nadella, R. K.; Viswanath, B.; Reddy, Y. C. J.; Jain, S.; & Anand, A. A Linkage and Exome Study Implicates Rare Variants of KANK4 and CAP2 in Bipolar Disorder in a Multiplex Family. *Bipolar Disord.* **2020**, *22*, 70; DOI:10.1111/bdi.12815
113. Masai, I.; Lele, Z.; Yamaguchi, M.; Komori, A.; Nakata, A.; Nishiwaki, Y.; Wada, H.; Tanaka, H.; Nojima, Y.; Hammerschmidt, M.; et al. N-Cadherin Mediates Retinal Lamination, Maintenance of Forebrain Compartments and Patterning of Retinal Neurites. *Development* **2003**, *130*, 2479 LP; DOI:10.1242/dev.00465
114. Zhang, W.; Mulieri, P. J.; Gaio, U.; Bae, G.-U.; Krauss, R. S.; & Kang, J.-S. Ocular Abnormalities in Mice Lacking the Immunoglobulin Superfamily Member Cdo. *FEBS J.* **2009**, *276*, 5998; DOI:10.1111/j.1742-4658.2009.07310.x
115. Shi, Y.; Tu, Y.; Mecham, R. P.; & Bassnett, S. Ocular Phenotype of Fbn2-Null Mice. *Investig. Ophthalmol. Vis. Sci.* **2013**, *54*, 7163; DOI:10.1167/iovs.13-12687
116. Chen, S.; Li, H.; Gaudenz, K.; Paulson, A.; Guo, F.; Trimble, R.; Peak, A.; Seidel, C.; Deng, C.; Furuta, Y.; et al. Defective FGF Signaling Causes Coloboma Formation and Disrupts Retinal Neurogenesis. *Cell Res.* **2013**, *23*, 254; DOI:10.1038/cr.2012.150
117. Fotaki, V.; Smith, R.; Pratt, T.; & Price, D. J. Foxg1 Is Required to Limit the Formation of Ciliary Margin Tissue and Wnt/β-Catenin Signalling in the Developing Nasal Retina of the Mouse. *Dev. Biol.* **2013**, *380*, 299; DOI:10.1016/j.ydbio.2013.04.017
118. Cai, Z.; Tao, C.; Li, H.; Ladher, R.; Gotoh, N.; Feng, G. S.; Wang, F.; & Zhang, X. Deficient FGF Signaling Causes Optic Nerve Dysgenesis and Ocular Coloboma. *Dev.* **2013**, *140*, 2711; DOI:10.1242/dev.089987
119. Thompson, B.; Chen, Y.; Philippe, J.; Anderson, D.; Prakash, J. G.; Davidson, E.; Apostolopoulos, N.; Schey, K.; Katsanis, N.; Orlicky, D. J.; et al. <Em>Gclc</Em> Deletion in Surface-Ectoderm Tissues Induces Microphthalmia. *BioRxiv* **2019**, 700591; DOI:10.1101/700591
120. Lee, H. Y.; Wroblewski, E.; Philips, G. T.; Stair, C. N.; Conley, K.; Reedy, M.; Mastick, G. S.; & Brown, N. L. Multiple Requirements for Hes1 during Early Eye Formation. *Dev. Biol.* **2005**, *284*, 464; DOI:https://doi.org/10.1016/j.ydbio.2005.06.010
121. Roscioli, T.; Kamsteeg, E. J.; Buysse, K.; Maystadt, I.; Van Reeuwijk, J.; Van Den Elzen, C.; Van Beusekom, E.; Riemersma, M.; Pfundt, R.; Vissers, L. E. L. M.; et al. Mutations in ISPD Cause Walker-Warburg Syndrome and Defective Glycosylation of α-Dystroglycan. *Nat. Genet.* **2012**, *44*, 581; DOI:10.1038/ng.2253
122. Xue, Y.; Gao, X.; Lindsell, C. E.; Norton, C. R.; Chang, B.; Hicks, C.; Gendron-Maguire, M.; Rand, E. B.; Weinmaster, G.; & Gridley, T. Embryonic Lethality and Vascular Defects in Mice Lacking the Notch Ligand Jagged1. *Hum. Mol. Genet.* **1999**, *8*, 723; DOI:10.1093/hmg/8.5.723
123. Gross, J. M. & Perkins, B. D. *Zebrafish Mutants as Models for Congenital Ocular Disorders in Humans*, Molecular Reproduction and

Development; DOI:10.1002/mrd.20831
124. Weiss, O.; Kaufman, R.; Michaeli, N.; & Inbal, A. Abnormal Vasculature Interferes with Optic Fissure Closure in Lmo2 Mutant Zebrafish Embryos. *Dev. Biol.* **2012**, *369*, 191; DOI:https://doi.org/10.1016/j.ydbio.2012.06.029
125. Gregory-Evans, C. Y. Ocular Coloboma: A Reassessment in the Age of Molecular Neuroscience. *J. Med. Genet.* **2004**, *41*, 881; DOI:10.1136/jmg.2004.025494
126. Kim, T. H.; Goodman, J.; Anderson, K. V.; & Niswander, L. Phactr4 Regulates Neural Tube and Optic Fissure Closure by Controlling PP1-, Rb-, and E2F1-Regulated Cell-Cycle Progression. *Dev. Cell* **2007**, *13*, 87; DOI:10.1016/j.devcel.2007.04.018
127. Hughes, J. J.; Alkhunaizi, E.; Kruszka, P.; Pyle, L. C.; Grange, D. K.; Berger, S. I.; Payne, K. K.; Masser-Frye, D.; Hu, T.; Christie, M. R.; et al. Loss-of-Function Variants in PPP1R12A: From Isolated Sex Reversal to Holoprosencephaly Spectrum and Urogenital Malformations. *Am. J. Hum. Genet.* **2020**, *106*, 121; DOI:10.1016/j.ajhg.2019.12.004
128. Holly, V. L.; Widen, S. A.; Famulski, J. K.; & Waskiewicz, A. J. Sfrp1a and Sfrp5 Function as Positive Regulators of Wnt and BMP Signaling during Early Retinal Development. *Dev. Biol.* **2014**, *388*, 192; DOI:10.1016/j.ydbio.2014.01.012
129. Zhang, R.; Huang, H.; Cao, P.; Wang, Z.; Chen, Y.; & Pan, Y. Sma- and Mad-Related Protein 7 (Smad7) Is Required for Embryonic Eye Development in the Mouse. *J. Biol. Chem.* **2013**, *288*, 10275; DOI:10.1074/jbc.M112.416719
130. Wen, W.; Pillai-Kastoori, L.; Wilson, S. G.; & Morris, A. C. Sox4 Regulates Choroid Fissure Closure by Limiting Hedgehog Signaling during Ocular Morphogenesis. *Dev. Biol.* **2015**,; DOI:10.1016/j.ydbio.2014.12.026
131. Pillai-Kastoori, L.; Wen, W.; Wilson, S. G.; Strachan, E.; Lo-Castro, A.; Fichera, M.; Musumeci, S. A.; Lehmann, O. J.; & Morris, A. C. Sox11 Is Required to Maintain Proper Levels of Hedgehog Signaling during Vertebrate Ocular Morphogenesis. *PLOS Genet.* **2014**, *10*, e1004491
132. Wurm, A.; Sock, E.; Fuchshofer, R.; Wegner, M.; & Tamm, E. R. Anterior Segment Dysgenesis in the Eyes of Mice Deficient for the High-Mobility-Group Transcription Factor Sox11. *Exp. Eye Res.* **2008**, *86*, 895; DOI:https://doi.org/10.1016/j.exer.2008.03.004
133. Knickmeyer, M. D.; Mateo, J. L.; Eckert, P.; Roussa, E.; Rahhal, B.; Zuniga, A.; Krieglstein, K.; Wittbrodt, J.; & Heermann, S. TGFb-Facilitated Optic Fissure Fusion and the Role of Bone Morphogenetic Protein Antagonism. *Open Biol.* **2018**, *8*; DOI:10.1098/rsob.170134
134. Gasperowicz, M.; Surmann-Schmitt, C.; Hamada, Y.; Otto, F.; & Cross, J. C. The Transcriptional Co-Repressor TLE3 Regulates Development of Trophoblast Giant Cells Lining Maternal Blood Spaces in the Mouse Placenta. *Dev. Biol.* **2013**, *382*, 1; DOI:10.1016/j.ydbio.2013.08.005
135. Raca, G.; Jackson, C.; Warman, B.; Bair, T.; & Schimmenti, L. A. Next Generation Sequencing in Research and Diagnostics of Ocular Birth Defects. *Mol. Genet. Metab.* **2010**, *100*, 184; DOI:10.1016/j.ymgme.2010.03.004
136. Wyatt, A. W.; Osborne, R. J.; Stewart, H.; & Ragge, N. K. Bone Morphogenetic Protein 7 (BMP7) Mutations Are Associated with Variable Ocular, Brain, Ear, Palate, and Skeletal Anomalies. *Hum. Mutat.* **2010**, *31*, 781; DOI:10.1002/humu.21280
137. Hocking, J. C.; Famulski, J. K.; Yoon, K. H.; Widen, S. A.; Bernstein, C. S.; Koch, S.; Weiss, O.; Agarwala, S.; Inbal, A.; Lehmann, O. J.; et al. Morphogenetic Defects Underlie Superior Coloboma, a Newly Identified Closure Disorder of the Dorsal Eye. *PLoS Genet.* **2018**, *14*, 1; DOI:10.1371/journal.pgen.1007246
138. Gallardo, V. & Bovolenta, P. Positive and Negative Regulation of Shh Signalling in Vertebrate Retinal Development. *F1000Research* **2018**, *7*, F1000 Faculty Rev; DOI:10.12688/f1000research.16190.1
139. Robb, E. A.; Antin, P. B.; & Delany, M. E. Defining the Sequence Elements and Candidate Genes for the Coloboma Mutation. *PLoS One* **2013**, *8*; DOI:10.1371/journal.pone.0060267
140. Ragge, N. K.; Brown, A. G.; Poloschek, C. M.; Lorenz, B.; Henderson, R. A.; Clarke, M. P.; Russell-Eggitt, I.; Fielder, A.; Gerrelli, D.; Martinez-Barbera, J. P.; et al. Heterozygous Mutations of OTX2 Cause Severe Ocular Malformations. *Am. J. Hum. Genet.* **2005**, *76*, 1008; DOI:10.1086/430721
141. Prokudin, I.; Simons, C.; Grigg, J. R.; Storen, R.; Kumar, V.; Phua, Z. Y.; Smith, J.; Flaherty, M.; Davila, S.; & Jamieson, R. V. Exome Sequencing in Developmental Eye Disease Leads to Identification of Causal Variants in GJA8, CRYGC, PAX6 and CYP1B1. *Eur. J. Hum. Genet.* **2014**, *22*, 907; DOI:10.1038/ejhg.2013.268
142. Leung, G. K. C.; Mak, C. C. Y.; Fung, J. L. F.; Wong, W. H. S.;

Tsang, M. H. Y.; Yu, M. H. C.; Pei, S. L. C.; Yeung, K. S.; Mok, G. T. K.; Lee, C. P.; et al. Identifying the Genetic Causes for Prenatally Diagnosed Structural Congenital Anomalies (SCAs) by Whole-Exome Sequencing (WES). *BMC Med. Genomics* **2018**, *11*, 93; DOI:10.1186/s12920-018-0409-z
143. Tekendo-ngongang, C. & Muenke, M. Holoprosencephaly Overview 1 . Clinical Characteristics of Holoprosencephaly. **2020**, 1
144. Noh, H.; Lee, H.; Park, E.; & Park, S. Proper Closure of the Optic Fissure Requires Ephrin A5-EphB2-JNK Signaling. *Development* **2016**, *143*, 461 LP; DOI:10.1242/dev.129478
145. Crossley, P. H.; Martinez, S.; Ohkubo, Y.; & Rubenstein, J. L. R. Coordinate Expression of Fgf8, Otx2, Bmp4, and Shh in the Rostral Prosencephalon during Development of the Telencephalic and Optic Vesicles. *Neuroscience* **2001**, *108*, 183; DOI:https://doi.org/10.1016/S0306-4522(01)00411-0
146. Nieuwenhuis, E. & Hui, C. Hedgehog Signaling and Congenital Malformations. *Clin. Genet.* **2004**, *67*, 193; DOI:10.1111/j.1399-0004.2004.00360.x
147. Furimsky, M. & Wallace, V. A. Complementary Gli Activity Mediates Early Patterning of the Mouse Visual System. *Dev. Dyn.* **2006**, *235*, 594; DOI:10.1002/dvdy.20658
148. Zhang, C.; Wu, P.; Wang, L.; Gao, J.; Huang, X.; & Jiang, Y. Bilateral Congenital Macular Coloboma and Cataract: A Case Report. *Medicine (Baltimore).* **2019**, *98*, e14803; DOI:10.1097/MD.0000000000014803
149. Sanyanusin, P.; Schimmenti, L. A.; McNoe, L. A.; Ward, T. A.; Pierpont, M. E. M.; Sullivan, M. J.; Dobyns, W. B.; & Eccles, M. R. Mutation of the PAX2 Gene in a Family with Optic Nerve Colobomas, Renal Anomalies and Vesicoureteral Reflux. *Nat. Genet.* **1995**, *9*, 358; DOI:10.1038/ng0495-358
150. Ufartes, R.; Schwenty-Lara, J.; Freese, L.; Neuhofer, C.; Möller, J.; Wehner, P.; van Ravenswaaij-Arts, C. M. A.; Wong, M. T. Y.; Schanze, I.; Tzschach, A.; et al. Sema3a Plays a Role in the Pathogenesis of CHARGE Syndrome. *Hum. Mol. Genet.* **2018**, *27*, 1343; DOI:10.1093/hmg/ddy045
151. Adly, N.; Alhashem, A.; Ammari, A.; & Alkuraya, F. S. Ciliary Genes TBC1D32/C6orf170 and SCLT1 Are Mutated in Patients with OFD Type IX. *Hum. Mutat.* **2014**, *35*, 36; DOI:https://doi.org/10.1002/humu.22477
152. El Chehadeh, S.; Kerstjens-Frederikse, W. S.; Thevenon, J.; Kuentz, P.; Bruel, A.-L.; Thauvin-Robinet, C.; Bensignor, C.; Dollfus, H.; Laugel, V.; Rivière, J.-B.; et al. Dominant Variants in the Splicing Factor PUF60 Cause a Recognizable Syndrome with Intellectual Disability, Heart Defects and Short Stature. *Eur. J. Hum. Genet.* **2017**, *25*, 43; DOI:10.1038/ejhg.2016.133
153. Vervoort, V. S. Sorting Nexin 3 (SNX3) Is Disrupted in a Patient with a Translocation t(6;13)(Q21;Q12) and Microcephaly, Microphthalmia, Ectrodactyly, Prognathism (MMEP) Phenotype. *J. Med. Genet.* **2002**, *39*, 893; DOI:10.1136/jmg.39.12.893
154. James, A.; Lee, C.; Williams, A. M.; Angileri, K.; Lathrop, K. L.; & Gross, J. M. The Hyaloid Vasculature Facilitates Basement Membrane Breakdown during Choroid Fissure Closure in the Zebrafish Eye. *Dev. Biol.* **2016**, *419*, 262; DOI:https://doi.org/10.1016/j.ydbio.2016.09.008
155. Khorram, D.; Choi, M.; Roos, B. R.; Stone, E. M.; Kopel, T.; Allen, R.; Alward, W. L. M.; Scheetz, T. E.; & Fingert, J. H. Novel TMEM98 Mutations in Pedigrees with Autosomal Dominant Nanophthalmos. *Mol. Vis.* **2015**, *21*, 1017
156. Pineda-Alvarez, D. E.; Solomon, B. D.; Roessler, E.; Balog, J. Z.; Hadley, D. W.; Zein, W. M.; Hadsall, C. K.; Brooks, B. P.; & Muenke, M. A Broad Range of Ophthalmologic Anomalies Is Part of the Holoprosencephaly Spectrum. *Am. J. Med. Genet. Part A* **2011**, *155*, 2713; DOI:10.1002/ajmg.a.34261
157. Brown, J. D.; Dutta, S.; Bharti, K.; Bonner, R. F.; Munson, P. J.; Dawid, I. B.; Akhtar, A. L.; Onojafe, I. F.; Alur, R. P.; Gross, J. M.; et al. Expression Profiling during Ocular Development Identifies 2 Nlz Genes with a Critical Role in Optic Fissure Closure. *Proc. Natl. Acad. Sci.* **2009**, *106*, 1462; DOI:10.1073/pnas.0812017106

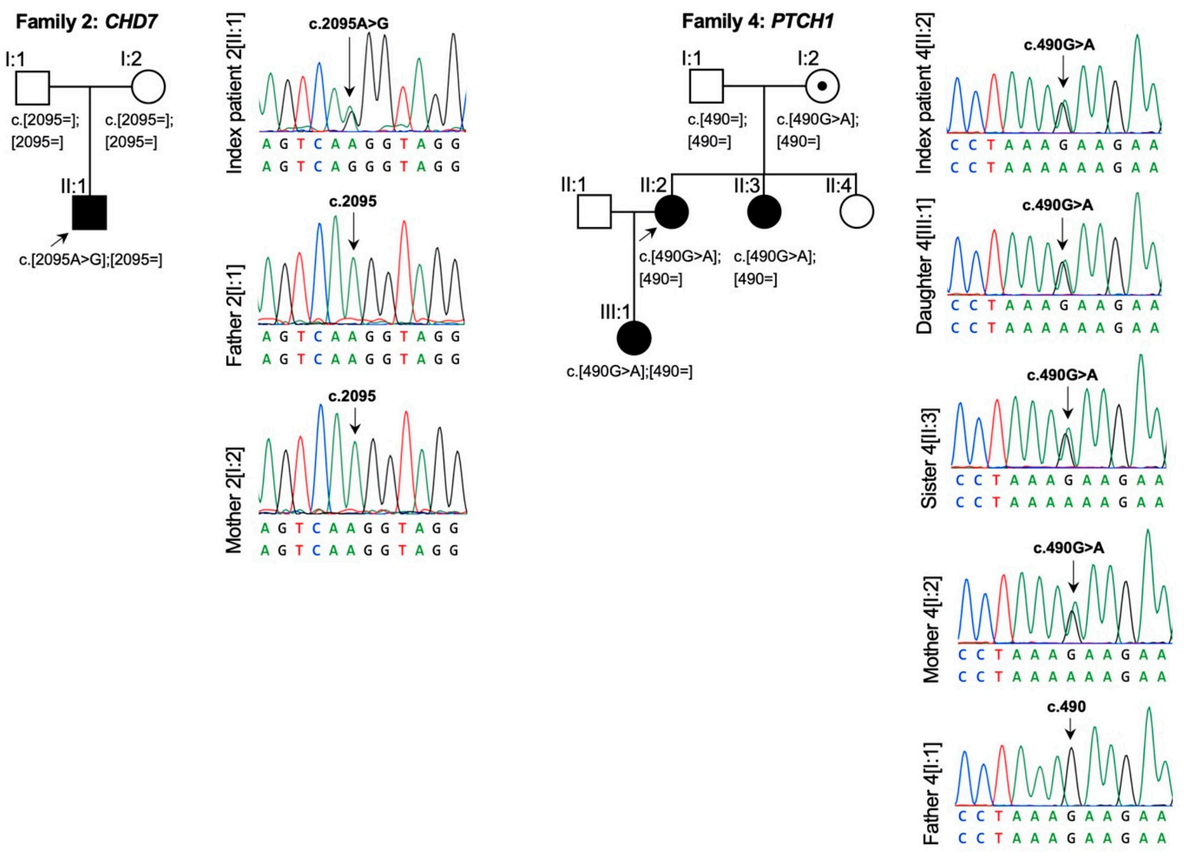

Figure S1: Pedigrees and Sanger sequencing electropherograms of families 2 and 4.

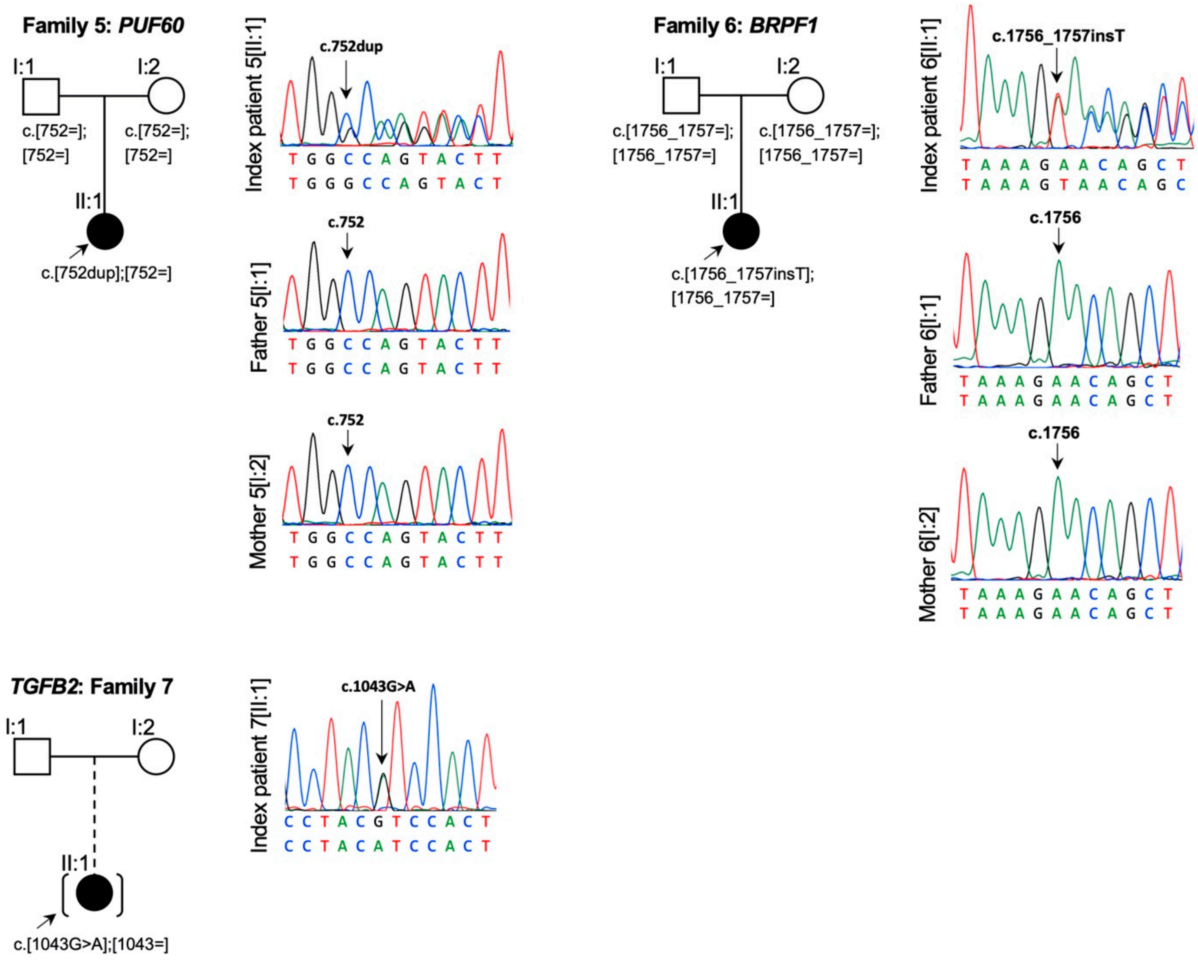

Figure S2: Pedigrees and Sanger sequencing electropherograms of families 5-7.
